# A Ratiometric Fluorescence Method Based on PCN-224-DABA for the Detection of Se(IV) and Fe(III)

**DOI:** 10.3390/bios14120626

**Published:** 2024-12-19

**Authors:** Mao-Ling Luo, Guo-Ying Chen, Wen-Jia Li, Jia-Xin Li, Tong-Qing Chai, Zheng-Ming Qian, Feng-Qing Yang

**Affiliations:** 1 School of Chemistry and Chemical Engineering, Chongqing University, Chongqing 401331, China; 20185486@cqu.edu.cn (M.-L.L.); 20221801017@stu.cqu.edu.cn (G.-Y.C.); 202218021016@stu.cqu.edu.cn (J.-X.L.); 20175531@cqu.edu.cn (T.-Q.C.); 2 Dongguan HEC Cordyceps R&D Co., Ltd., Dongguan 523850, China; liwenjia@hec.cn

**Keywords:** ratiometric fluorescence, quenching, selenium, ferric iron, porphyrin metal–organic framework

## Abstract

In this study, 3,4-diaminobenzoic acid (DABA) was introduced into the porphyrin metal–organic framework (PCN-224) for the first time to prepare a ratiometric fluorescent probe (PCN-224-DABA) to quantitatively detect ferric iron (Fe(III)) and selenium (IV) (Se(IV)). The fluorescence attributed to the DABA of PCN-224-DABA at 345 nm can be selectively quenched by Fe(III) and Se(IV), but the fluorescence emission peak attributed to tetrakis (4-carboxyphenyl) porphyrin (TCPP) at 475 nm will not be disturbed. Therefore, the ratio of I_345nm_/I_475nm_ with an excitation wavelength of 270 nm can be designed to determine Fe(III) and Se(IV). After the experimental parameters were systematically optimized, the developed method shows good selectivity and interference resistance for Fe(III) and Se(IV) detection, and has good linearity in the ranges of 0.01–4 μM and 0.01–15 μM for Fe(III) and Se(IV) with a limit of detection of 0.045 μM and 0.804 μM, respectively. Furthermore, the quenching pattern was investigated through the Stern–Volmer equation, and the results suggest that both Se(IV) and Fe(III) quenched on PCN-224-DABA can be attributed to the dynamic quenching. Finally, the constructed ratiometric fluorescent probe was applied in the spiked detection of lake water samples, which shows good applicability in real sample analysis. Moreover, the Fe(III) and Se(IV) contents in spinach and selenium-enriched rice were determined, respectively.

## 1. Introduction

Selenium (Se) is an indispensable trace element that is relevant to the human metabolism [1,2], and which is presented in the human body as selenomethionine and selenocystine (Se-Cys). Furthermore, selenomethionine and Se-Cys are involved in the composition of selenoproteins [3], which are mainly classified as glutathione peroxidase and thioredoxin reductase, significantly protecting against oxidative damage and regulating intracellular redox status and thyroid hormone metabolism [4]. In soil and water, Se exists mainly in the inorganic forms of selenate (SeO_4_^2−^, Se(VI)) and selenite (SeO_3_^2−^, Se(IV)), which can be converted to Se-Cys again by plant uptake via the assimilatory sulfate reduction pathway for its similarity to sulfate [5]. Notably, despite the fact that Se intake in trace amounts benefits human health, the intake limit falls in a narrow range between deficit and toxicity [4]. Accordingly, the World Health Organization (WHO) states that human Se intake should be within the range of between 40 μg/day and 400 μg/day [6]. Relevant reports have demonstrated that exposure to elevated levels of Se promotes the generation of reactive oxygen species (ROS), induces cellular damage, and triggers apoptosis, resulting in skin discoloration and hair and nail loss [3,7]. Iron (Fe) is also an essential micronutrient that is engaged in various essential metabolic processes in the body, including hematopoiesis and mitochondrial electron transport [8]. Inadequate intake or malabsorption of Fe can lead to iron deficiency anemia, while an excessive concentration or imbalance of iron is associated with endocrine disorders, cancer, and neurological diseases [9,10,11]. On the other hand, the notable rise in industrial activities has resulted in the accumulation of persistent metallic pollutants in water, especially Fe(III), adversely affecting aquatic organisms and other biological communities. The European Union (EU) demands that the Fe(III) concentration in drinking water should not exceed 3.57 µmol/L [12].

Currently, the developed detection techniques for Se and Fe are mainly atomic absorption/emission spectroscopy (AAS/AES) [13,14,15], atomic fluorescence spectroscopy (AFS) [16,17], high-performance liquid chromatography (HPLC) [18,19], ion chromatography (IC) [20,21], inductively coupled plasma–mass spectrometer (ICP-MS) [22,23,24], electrochemistry [25,26,27], colorimetry [28,29], and fluorescence spectroscopy [30,31,32]. Although some of the above detection methods are extremely sensitive for ion quantification, large and expensive instruments, specialized operators, and complicated sample pre-treatment processes limit their applications in the rapid detection of real samples. Therefore, it is essential to establish simple and efficient methods for detecting ions with high sensitivity. Among the aforementioned detection techniques, fluorescence spectroscopy-based methods have garnered significant attention for their ease of handling and superior sensitivity compared to colorimetric approaches.

Porphyrin-based metal–organic frameworks (MOFs) are emerging as luminescent MOF (LMOF) materials for the fabrication of fluorescent sensing platforms [33,34,35]. In particular, PCN-224 is a prototypical porphyrin Zr-MOF material synthesized through linking a stabilized luminescent center TCPP as a ligand with a Zr_6_ cluster via a carboxyl group [36], which is characterized by a simple structure, extremely high specific surface area, ease of modification, and abundant active sites [37,38]. Recently, PCN-224 has been designed for the quantitative determination of some ions. Moradi et al. synthesized PCN-224 as a fluorescent probe for the simultaneous selective determination of Cd^2+^ and Br^−^ as well as tetrahydrofuran (THF) small molecules, both of which showed good sensitivity [39]. However, the single fluorescence emission signal is susceptible to the influence of background fluorescence and environmental interference, resulting in poor selectivity and low precision. Based on the abundant active sites on the PCN-224 surface, Ma et al. utilized PCN-224 as a single-component ratiometric fluorescent probe to detect phosphate ions with a detection limit of 54 nM [40]. There are two fluorescence emission peaks at a certain excitation wavelength of the ratiometric fluorescent probe and the two signal peaks can be self-calibrated in the detection, which effectively improves the accuracy and selectivity of the measurement. Furthermore, LMOFs with dual-emission properties, such as PCN-224, can be further endowed with multi-emission properties through in situ modification [41], ion doping [42], and other methods to achieve highly specific determination of particular analytes.

**Figure 1:**
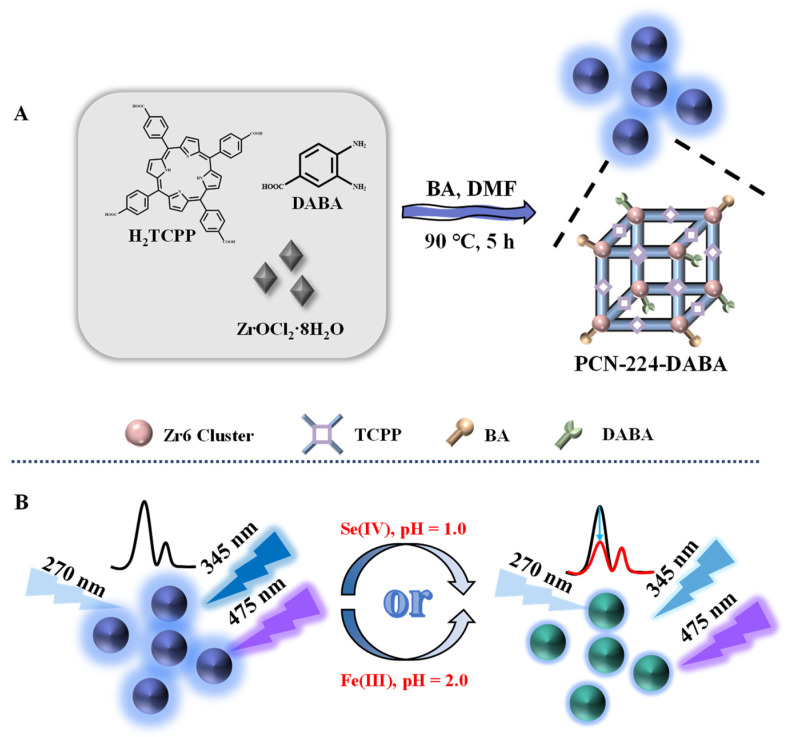
Schematic of the preparation of PCN-224-DABA and its applications in the determination of Se(IV) and Fe(III).

In this study, the 3,4-diaminobenzoic acid (DABA) molecule was successfully embedded into the structure of PCN-224 through a one-pot method for the first time to engineer a ratiometric fluorescent probe (PCN-224-DABA) (Figure 1), which was utilized for the selectively quantitative detection of Fe(III) and Se(IV) in actual samples with high sensitivity. The synthesized PCN-224-DABA not only features the emission peak of DABA at an excitation wavelength of 270 nm, but also retains the fluorescence property of PCN-224. Under acidic conditions, the fluorescence emission peak (345 nm) of DABA in this probe can be significantly quenched by Fe(III) and Se(IV), but the fluorescence intensity (475 nm) of the TCPP ligand remains unaffected. Therefore, the ratio change in the fluorescence emission peaks at 345 nm and 475 nm (I_345nm_/I_475nm_) was chosen for the selective quantitative determination of Fe(III) and Se(IV) (λ_ex_ = 270 nm). The optimized molar ratio of the two luminescent molecules (TCPP and DABA) in PCN-224-DABA was determined, and the morphology as well as the surface functional groups were investigated. Subsequently, the amount of PCN-224-DABA, reaction pH, temperature, and reaction time for the detection of Fe(III) and Se(IV) were optimized, and their mechanisms of action were explored. Finally, the PCN-224-DABA ratiometric fluorescent probe was successfully applied in the quantitative detection of Fe(III) and Se(IV) in spinach, rice, and lake water, respectively. The fluorescent probe PCN-224-DABA developed in this study, which presents a novel approach for the selective detection of Se(IV) and Fe(III), contributes innovative concepts for the design of multi-emission-ratio fluorescent probes.

## 2. Materials and Methods

### 2.1. Materials and Reagents

Details of the reagents and instruments used in this study are summarized in Sections S1 and S2 of the Supplementary Materials.

### 2.2. Preparation of PCN-224-DABA

In brief, 150 mg of ZrOCl_2_·8H_2_O, 50 mg of TCPP, 69.2 mg of DABA, and 1.4 g of benzoic acid (BA) were mixed with 50 mL of DMF in a round-bottomed flask and stirred for 5 h at 90 °C in an oil bath. After completion of the reaction, centrifugation was performed at 13,000 rpm (11,400× *g*) for 30 min of the obtained material, which was then washed twice, sequentially, with DMF and water. Finally, the obtained PCN-224-DABA was suspended in 8 mL of ultrapure water and kept at 4 °C for storage in the dark. PCN-224 was synthesized with a similar procedure, but without the addition of DABA.

### 2.3. PCN-224-DABA-Based Ratiometric Fluorescence for Se(IV) Detection

In sequence, 100 μL of PCN-224-DABA (30-fold dilution of the original solution), 800 μL of Tris-HCl buffer with pH = 1.0, and 100 μL of Se(IV) standard solution were added to a 1.5 mL centrifuge tube, and the reaction was performed for 120 min at 80 °C in a water bath. Subsequently, the fluorescence emission spectra at 280–540 nm were measured at λ_ex_ = 270 nm, excitation/emission slit width = 10/10 nm, photomultiplier tube (PMT) voltage = 500 V, and scanning speed = 1200 nm/min.

### 2.4. PCN-224-DABA-Based Ratiometric Fluorescence for Fe(III) Detection

In brief, 100 μL of PCN-224-DABA (30-fold dilution of the stock solution), 800 μL of Tris-HCl buffer with pH = 2.0, and 100 μL of Fe(III) standard solution were mixed for 7 min at 60 °C in a water bath. Subsequently, fluorescence emission spectra at 280–540 nm were acquired (λ_ex_ = 270 nm, excitation/emission slit width = 10/10 nm, PMT voltage = 500 V, scanning speed = 1200 nm/min).

### 2.5. Selectivity and Interference Study

To evaluate the suitability of the assay for Fe(III) and Se(IV) detection in real samples, potential substances may be presented in the samples, including cations of Zn(II), Mn(II), Pb(II), Al(III), Mg(II), Na(I), Co(II), and Ca(II), acid ions of HAsO_4_^2−^, CO_3_^2−^, HCO_3_^−^, PO_4_^3−^, HPO_4_^2−^, and H_2_PO_4_^−^, amino acids of proline, leucine, glutamic acid, histidine, and valine, and nucleosides of cytidine, inosine, thymidine, thymine, uracil, adenosine, and uridine, which were chosen to examine the selectivity and interference resistance of the established method. There were 1 mM, 0.1 mM, and 0.05 mM concentrations of interfering ions, Se(IV), and Fe(III), respectively, and the detection procedures were the same as in Section 2.3 and Section 2.4, respectively.

### 2.6. Real Sample Analysis

Spinach and selenium-enriched rice were purchased from the local Yonghui supermarket and selenium-enriched food store, respectively. The methods for the extraction of Fe(III) and Se(IV) from spinach and selenium-enriched rice are described in Section S3 of the Supplementary Material. The lake water samples of Yun Lake and Jin Lake were taken from Chongqing University. They were filtered once with a filter membrane (0.22 μm) and then used for subsequent spiking experiments. In brief, 0.5, 1, and 3 μM of Fe(III) were spiked to the above-treated lake water samples and 1, 5, and 10 μM of Se(IV) were added to the treated selenium-enriched rice solution and lake water samples, respectively. Then, the recoveries and RSD values of spiked samples were measured as in Section 2.3 and Section 2.4 to assess the reliability of the assay for analyzing real samples.

## 3. Results and Discussion

### 3.1. Characterizations of PCN-224-DABA

**Figure 2:**
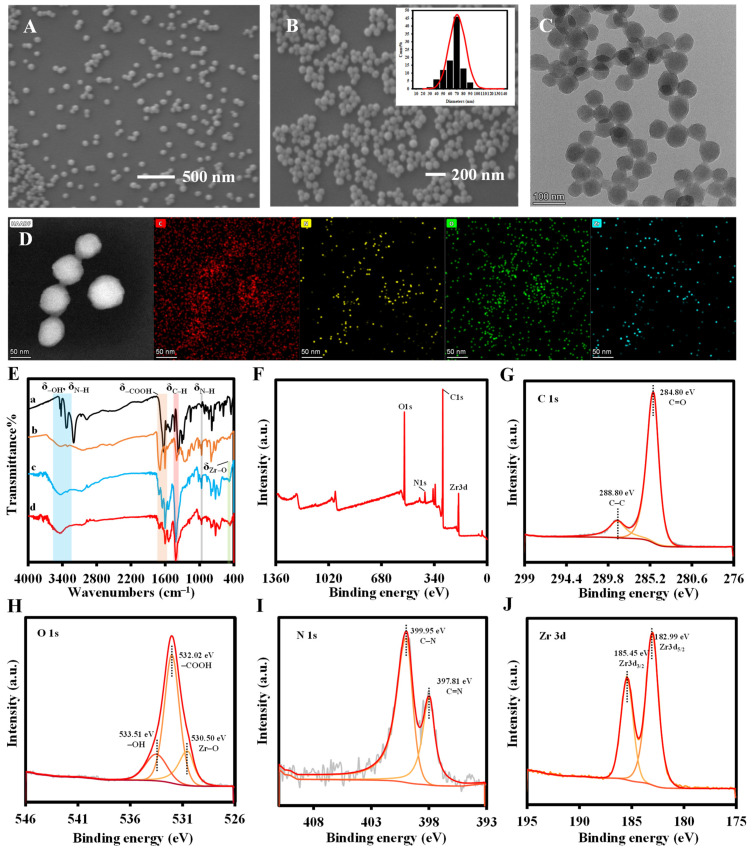
(**A**,**B**) SEM and (**C**) TEM images, and (**D**) the element mapping of PCN-224-DABA; (**E**) FT-IR spectra of (a) DABA, (b) TCPP, (c) PCN-224, and (d) PCN-224-DABA; XPS spectra of (**F**) PCN-224-DABA, (**G**) C 1s, (**H**) O 1s, (**I**) N 1s, and (**J**) Zr 3d.

PCN-224 and PCN-224-DABA with a TCPP to DABA molar ratio of 1:7 were characterized. In Figure S1 and Figure 2, the scanning electron microscopy (SEM) and transmission electron microscopy (TEM) images show spherical shapes and uniform distribution of the synthesized PCN-224 and PCN-224-DABA morphologies with average particle sizes of about 84 nm and 60 nm, respectively. Interestingly, the shape of PCN-224-DABA does not change with the decrease in the TCPP/DABA molar ratio, but the particle size is reduced (Figure S2). In addition, it is evident from the energy-dispersive X-ray spectroscopy (EDX) analysis of Figure S1D and Figure 2D that both the synthesized PCN-224 and PCN-224-DABA consist of the elements C, N, O, and Zr. High-resolution transmission microscopy (HRTEM) images indicate that both PCN-224 and PCN-224-DABA synthesized in this study are free of lattice fringes, which is consistent with XRD measurements, indicating that the synthesized materials are amorphous (Figure S3). Fourier transform infrared spectroscopy (FT-IR) spectra demonstrate the stretching vibrations of -OH, N-H, -COOH, and C-H in the structures of DABA and TCPP yielded characteristic absorption peaks at 3423 cm^−1^, 3332 cm^−1^, 1625 cm^−1^, and 1417 cm^−1^, respectively (curves a and b in Figure 2E) [43,44]. Furthermore, for PCN-224 and PCN-224-DABA, the coordination of the carboxyl group (-COOH) with the zirconium atom (Zr) resulted in a significant attenuation of the IR absorption at 1692 cm^−1^ (C=O) and a new absorption peak affiliated to Zr-O at 660 cm^−1^ [45], proving the successful preparation of PCN-224 and PCN-224-DABA (curves c and d in Figure 2E) [38,46]. The elemental composition and chemical structure of PCN-224-DABA were investigated by X-ray photoelectron spectroscopy (XPS) (Figure 2F), which agree with the EDX results that PCN-224-DABA contains the four elements of C, N, O, and Zr. The C 1s high-resolution XPS spectrum of Figure 2G shows two peaks at 284.80 eV and 288.80 eV, corresponding to C=O and C=C, respectively [47,48]. In Figure 2H, the peaks at 530.50 eV, 532.02 eV, and 533.51 eV in the O 1s spectrum are attributed to Zr-O, -COOH, and -OH, respectively [48]. These results reveal the successful loading of three ligand molecules as well as Zr atoms on the PCN-224-DABA. Furthermore, two peaks at 397.81 eV and 399.95 eV can be observed in the N 1s spectrum, which are associated with C-N and C=N, respectively (Figure 2I) [48], suggesting that the porphyrin ring and amino group are presented in PCN-224-DABA. In addition, it can be recognized that the two peaks of Zr 3d_3/2_ and Zr 3d_5/2_ are located at 185.45 eV and 182.99 eV, respectively, in the XPS spectrum of Zr 3d (Figure 2J), which proves that the valence state of Zr is +4 in PCN-224-DABA [47,48].

### 3.2. Feasibility and Detection Mechanism

**Figure 3:**
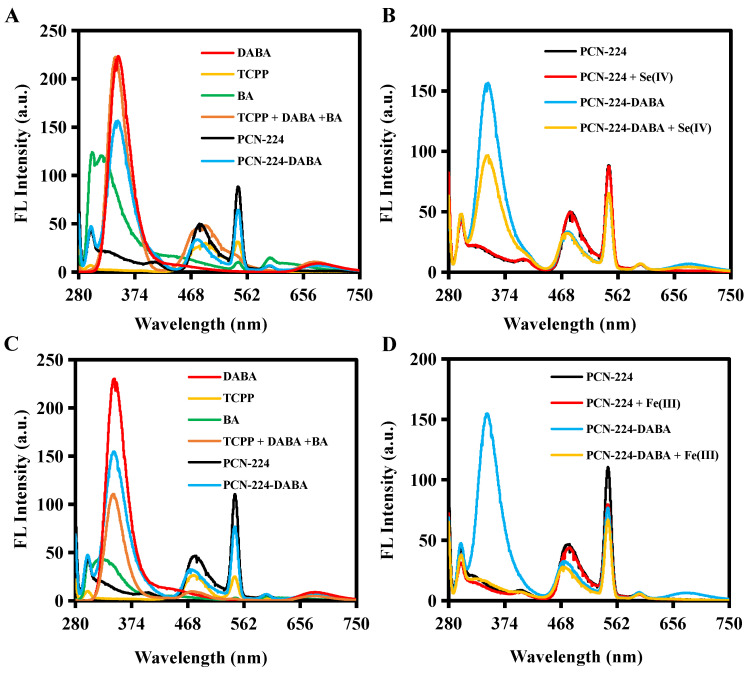
Fluorescence emission spectra of different systems at (**A**,**B**) pH = 1 and (**C**,**D**) pH = 2.

**Figure 4:**
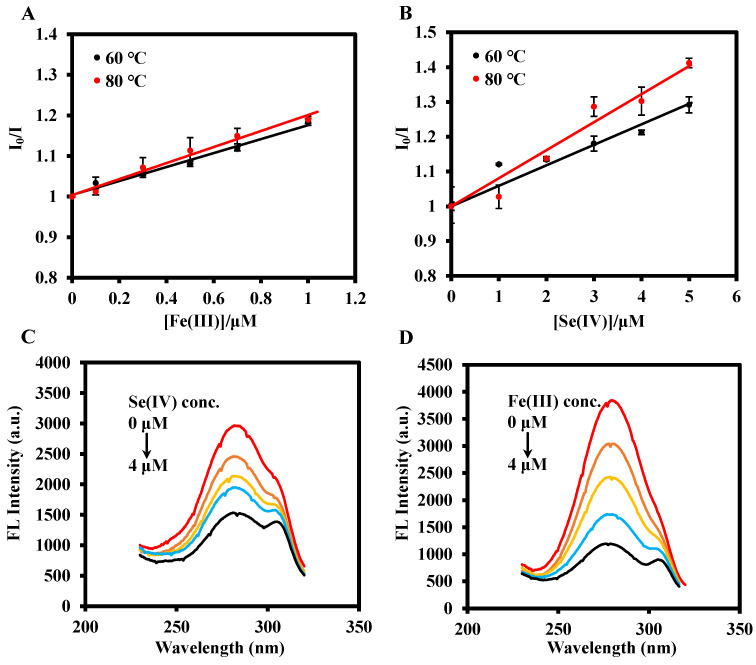
Stern–Volmer diagram of (**A**) PCN-224-DABA + Se(IV) and (**B**) PCN-224-DABA + Fe(III); emission spectrum of PCN-224-DABA with different concentrations of (**C**) Se(IV) and (**D**) Fe(III).

First, the excitation spectra of DABA and PCN-224-DABA indicated that their maximum excitation wavelength is 270 nm (Figure S4A). Furthermore, Figure 3A,C display the fluorescence emission spectra of different ligands, mixed ligands, PCN-224, and PCN-224-DABA at pH = 1 and pH = 2. The fluorescence emission peaks (λ_ex_ = 270 nm) of BA, DABA, and TCPP are located at 320 nm, 345 nm/685 nm, and 480 nm at pH = 1, and 320 nm, 345 nm/685 nm, and 475 nm at pH = 2, respectively. The synthesized PCN-224-DABA exhibited fluorescence emission peaks characteristic of both DABA and TCPP, suggesting the successful incorporation of DABA into the PCN-224 framework. Interestingly, the addition of Se(IV) or Fe(III) can induce a significant decrease in the fluorescence emission peak (345 nm) attributed to the DABA and PCN-224-DABA, but the fluorescence emission peak at 475 nm and UV/vis spectra of TCPP remains unchanged (Figure 3B,D and Figure S4B–D). Consequently, a ratiometric fluorescence method based on I_345nm_/I_475nm_ was constructed for Se(IV) or Fe(III) detection. To reduce the impact of the Rayleigh scattering peak (2λ_ex_) associated with the excitation light on detection, only the peaks of the emission spectrum in the range of 280–540 nm were subsequently documented. Moreover, the quenching mechanism was investigated utilizing the Stern–Volmer equation, which is I_0_/I = 1 + *K*_sv_[Q], where I_0_ and I denote the ratio values of I_345nm_/I_475nm_ in the absence and presence of Se(IV) or Fe(III), respectively, *K*_sv_ stands for the Stern–Volmer constant, and [Q] is the value of the different concentrations of Se(IV) or Fe(III). Figure 4A,B show the plots of the Stern–Volmer equation, and the slope (*K*_sv_) increases with the temperature, indicating a dynamic quenching [49]. In addition, the excitation spectrum of PCN-224-DABA exhibited minimal spectral alterations, with the exception of a reduction in the fluorescence intensity corresponding to increasing concentrations of Se(IV) and Fe(III) (Figure 4C,D), indicating that no basal complexes were formed, ruling out static quenching [50]. Therefore, the quenching of PCN-224-DABA by both Se(IV) and Fe(III) can be attributed to dynamic quenching.

**Figure 5:**
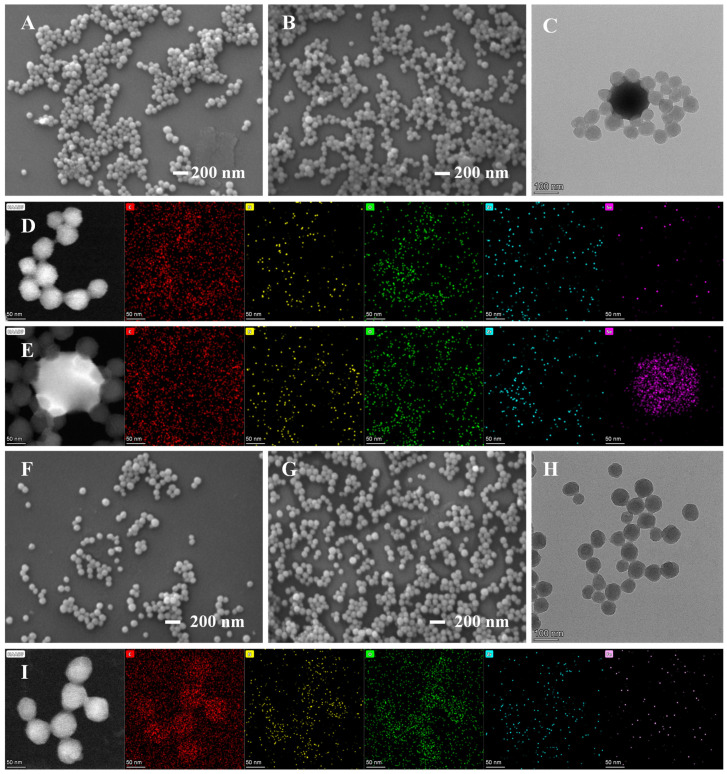
SEM images of PCN-224-DABA with (**A**) Tris-HCl (pH = 1), (**B**) Se(IV), (**F**) Tris-HCl (pH = 2.0), and (**G**) Fe(III); TEM images of PCN-224-DABA with (**C**–**E**) Se(IV) and (**H**,**I**) with Fe(III).

**Figure 6:**
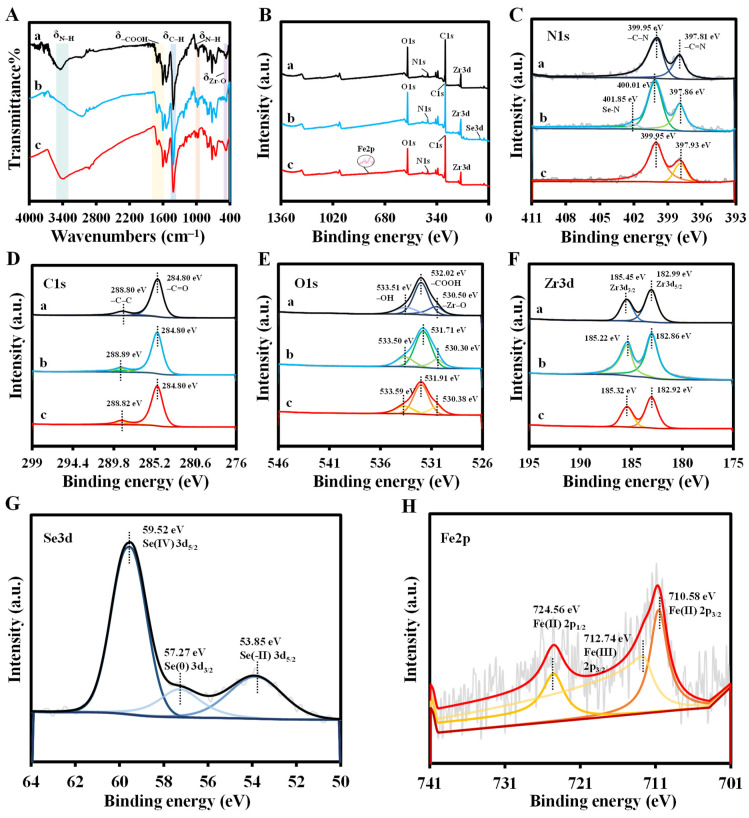
(**A**) FT-IR and (**B**) XPS spectra of PCN-224-DABA (a), PCN-224-DABA + Se(IV) (b), and PCN-224-DABA + Fe(III) (c); XPS spectra of (**C**) N 1s, (**D**) C 1s, (**E**) O 1s, and (**F**) Zr 3d of PCN-224-DABA (a), PCN-224-DABA + Se(IV) (b), and PCN-224-DABA + Fe(III) (c); XPS spectra of (**G**) Se 3d and (**H**) Fe 2p.

To conduct a more in-depth examination of the detection mechanism, the interaction of PCN-224-DABA with Se(IV) or Fe(III) was analyzed. The zeta potential of PCN-224-DABA demonstrates a positive charge potential at pH < 7 and a negative charge potential at pH > 7 with an isoelectric point of 6.429 (Figure S4E). The positive charge exhibited by PCN-224-DABA in acidic environments is likely attributed to the enrichment of functional amino groups on its surface. Interestingly, the zeta potential of PCN-224-DABA declined from 27.24 to 21.85 mV with the addition of Se(IV), and from 30.25 to 21.91 mV in the presence of Fe(III) (Figure S4F), suggesting that there is electron transfer between Se(IV)/Fe(III) and PCN-224-DABA. As shown in Figure 5, the presence of Se(IV) and Fe(III) and their corresponding detection conditions did not affect the morphology of PCN-224-DABA, indicating that the low pH buffer and the introduction of Se(IV) and Fe(III) do not have a significant effect on the structure of PCN-224-DABA. Furthermore, the EDX image of PCN-224-DABA (Figure 5D,E) after the addition of Se(IV) demonstrates that there is not only a small amount of Se elements distributed evenly on the surface of PCN-224-DABA, but also that some of the Se elements presented independently in spherical shapes. Considering that Se(IV) may be reduced in the assay, it is speculated that the center of the PCN-224-DABA aggregation in Figure 5C,E is composed of Se nanoparticles (SeNPs). In addition, the five elements of C, N, O, Zr, and Fe are presented in the PCN-224-DABA + Fe(III) system (Figure 5I). The FT-IR spectra show that the N-H characteristic peak (3332 cm^−1^) of PCN-224-DABA disappeared and that the stretching vibrational peak of N-H at 965 cm^−1^ was weakened with the addition of Se(IV) (Figure 6A), which is consistent with the appearance of a new peak (401.85 eV) attributed to Se-N in the N 1s spectrum (Figure 6C), suggesting that the Se can interact with the amino group of the o-phenylenediamine-like structure of PCN-224-DABA. The XPS spectra of Se(IV)-treated PCN-224-DABA display five elements, including C, N, O, Zr, and Se, in Figure 6B. The Se3d XPS spectrum shows three peaks belonging to Se(-II) (53.85 eV), Se(0) (57.27 eV), and Se(IV) (59.52 eV), respectively, [51] (Figure 6G), evidencing that a redox reaction occurs during the detection of Se(IV) (Se(IV)→Se(0), Se(-II)), echoing the phenomenon of TEM in Figure 5E. Similarly, three peaks located at 710.58 eV, 712.74 eV, and 724.56 eV can be observed in the Fe2p XPS spectra (Figure 6H), which represent Fe(II) 2p_3/2_, Fe(III) 2p_3/2_, and Fe(II) 2p_5/2_, respectively [52], signifying that the detection of Fe(III) can also be affiliated to the redox reaction. The redox reaction may be associated with the interaction of Fe(III) with the amino group, as evidenced by the weakening and shifting of the N-H stretching vibration peaks at 965 cm^−1^ and 3334 cm^−1^ in Figure 6A.

### 3.3. Optimization of Se(IV) and Fe(III) Detection Conditions

**Figure 7:**
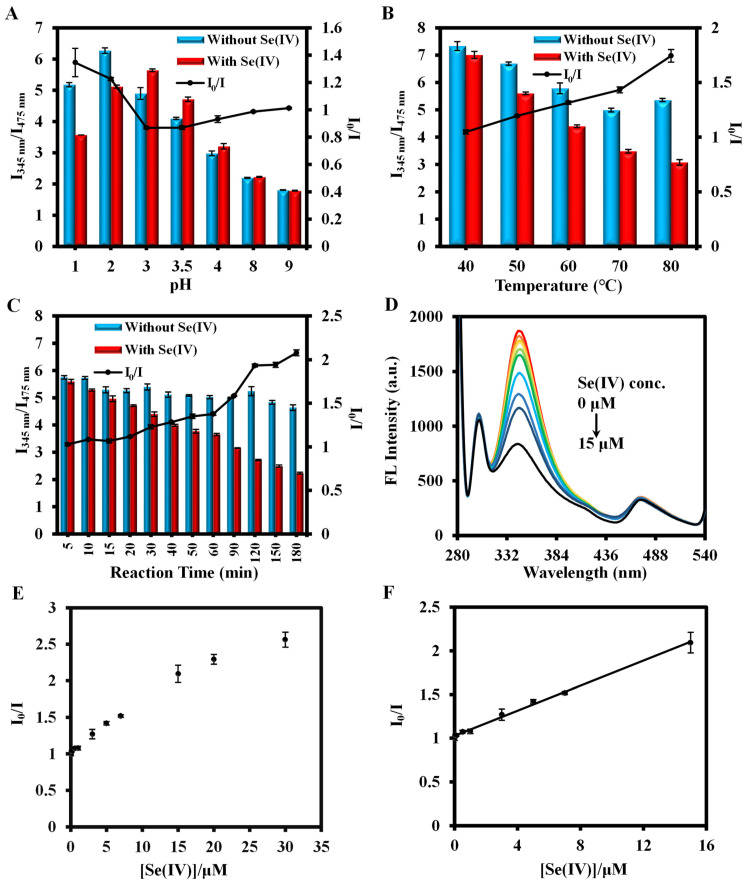
The effects of (**A**) pH, (**B**) temperature, and (**C**) reaction time on the detection of Se(IV); (**D**) fluorescence spectra of the reaction system with different concentrations of Se(IV) and (**E**,**F**) corresponding scatter plots.

**Figure 8:**
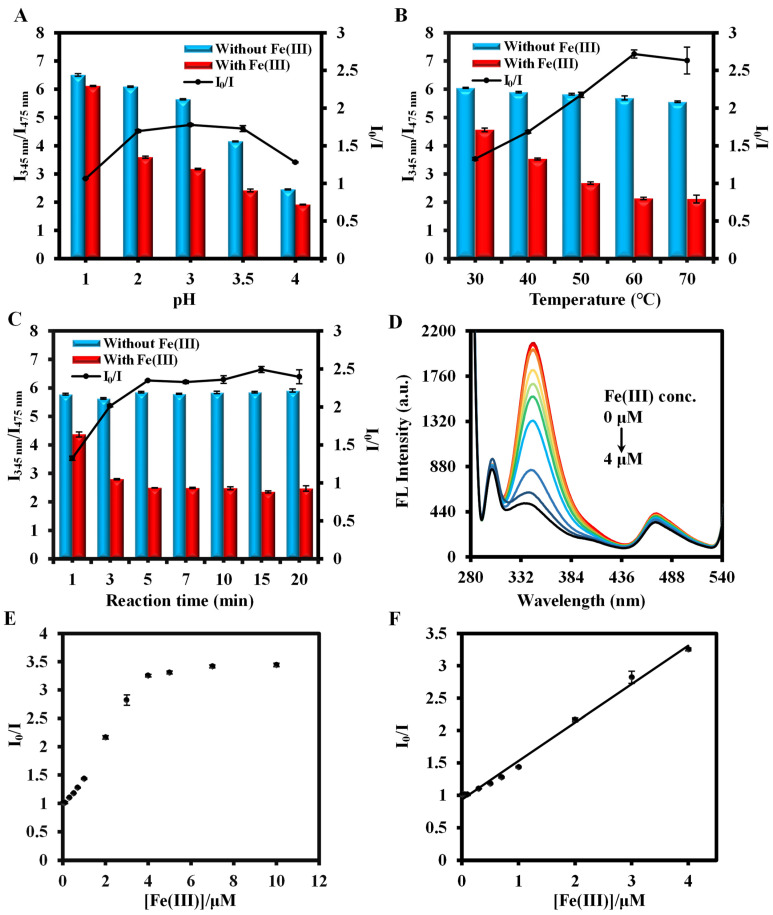
The effects of (**A**) pH, (**B**) temperature, and (**C**) reaction time on the detection of Fe(III); (**D**) fluorescence spectra of the reaction system with different concentrations of Fe(III) and (**E**,**F**) corresponding scatter plots.

Since the detection procedure is a quenching process, the magnitude of the ratio fluorescence value of I_345nm_/I_475nm_ was used as a criterion for the optimization. Figure S5A,B show that the ratio of I_345nm_/I_475nm_ increases as the molar ratio of TCPP to DABA decreases, reaching an optimized value at TCPP/DABA = 1/7. In addition, when the amount of material was fixed at 100 μL, the PCN-224-DABA stock solution to be diluted 30 times was selected (Figure S5C). Then, the effect of pH on the assay was explored. It can be observed from Figure S6A that the fluorescence emission peak for the DABA ligand changes depending on the pH level. At pH < 4, the fluorescence emission peak appears at 345 nm with an excitation light of 270 nm, whereas at pH ≥ 4, the emission peak red-shifts to 490 nm and the fluorescence intensity increases with the increase in pH. For PCN-224, its fluorescence emission is located at 475 nm at pH < 3, and the emission peak is blue-shifted to 450 nm at pH ≥ 3 (Figure S6B). In addition, the fluorescence spectrograms of PCN-224-DABA at different pH conditions are shown in Figure S6C, and the change rule of dual-wavelength emission positions is consistent with that of DABA and PCN-224 mentioned above. Moreover, the red-shift of the DABA and the blue-shift of TCPP fluorescence emission peaks make the two fluorescence emission peaks overlap, resulting in the inability to observe the dual-emission peaks in the spectrograms at pH values of 5, 6, and 7. On the other hand, PCN-224 showed enhanced fluorescence in alkaline environments (pH ≥ 8) [53], which facilitated the emergence of a fluorescence emission peak at 450 nm that was not obscured by DABA, allowing the dual-emission peaks to reappear. Therefore, the influence of pH on the assay was only evaluated at pH ≤ 4 and pH = 8 and 9. In addition, since the fluorescence intensity exceeded the instrument maximum detection range at pH ≥ 7, the instrumental setup conditions for PCN-224-DABA fluorescence emission at pH = 7, 8, and 9 in Figure S6C were changed to excitation/emission slit width = 5/10 nm, PMT voltage = 500 V, and scanning speed = 1200 nm/min. Based on the comparison of the value of I_0_/I in the presence or absence of Se(IV) or Fe(III), pH = 1 and pH = 2 were selected for Se(IV) and Fe(III) detection (Figure 7A and Figure 8A), respectively. The temperature in the assay was also a major influencing factor, and the final results are given in Figure 7B and Figure 8B. Although the value of I_0_/I increased with increasing temperature, 80 °C was finally picked as the detection temperature of Se(IV) for the considerations of experimental safety and operational stability. For Fe(III) detection, I_0_/I was optimized at a reaction temperature of 60 °C. Finally, there was an examination of the effect of reaction time on the detection. Figure 7C and Figure 8C indicate that Se(IV) and Fe(III) reacted completely at 120 min and 7 min, respectively. In summary, a reaction of 80 °C for 120 min at pH = 1 was selected for detecting Se(IV), and a reaction of 60 °C for 7 min at pH = 2 was chosen for Fe(III) detection. Furthermore, it is worth noting that PCN-224-DABA can maintain a good fluorescence performance for 20 days under either Se(IV) or Fe(III) detection conditions (Figure S6D).

### 3.4. Detection of Se(IV) and Fe(III) by the Ratiometric Fluorescence Method Based on PCN-224-DABA

Based on PCN-224-DABA, the fluorescence emission peaks attributed to DABA at 345 nm and TCPP at 475 nm were identified for the development of a ratiometric fluorescence method. The incorporation of Se(IV) causes the fluorescence quenching of DABA at 345 nm, which can be ascribed to the following reasons. (1) Se and the o-phenylenediamine group in the molecular structure of DABA can form a five-membered cyclic Se-DABA complex, and the light-induced electron transfer phenomenon occurs in the ring [54]; (2) the redox reaction between Se(IV) and DABA changes the molecular structure and the electron arrangement of DABA. However, there is no change in the fluorescence intensity at 475 nm with the addition of different concentrations of Se(IV). Therefore, a ratiometric fluorescence method was constructed based on the ratio of I_345nm_/I_475nm_ for the quantitative determination of Se(IV).

As shown in Figure 7D–F, Se(IV) exhibits good linearity in the 0.01–15 μM concentration range with a calibration curve of I_0_/I = 1 + 0.0745[Se(IV)] (R^2^ = 0.9967) and a limit of detection (LOD) of 0.804 μM (LOD = 3*σ*/S, where S is the slope of the calibration curve and *σ* is an 11-group standard deviation of the blanks). Although there is an order of magnitude increase in sensitivity compared to DABA alone (Figure S7), the detection range is still narrower than the other methods (Table 1), which may be related to the limit of fluorescence quenching. Similar to the detection principle of Se(IV), Fe(III) can also undergo a redox reaction with the amino group on DABA in PCN-224-DABA, attenuating fluorescence at 345 nm. The linear regression equation for Fe(III) detection at the concentration of 0.01–4 μM was plotted as I_0_/I = 1 + 0.5694[Fe(III)] (R^2^ = 0.9933), and the LOD value is 0.045 μM (Figure 8F), which is below the qualifying standard of Fe(III) in water as stipulated by the WHO and EPA (Table 2) [12,55].

### 3.5. Selectivity and Interference Study

**Figure 9:**
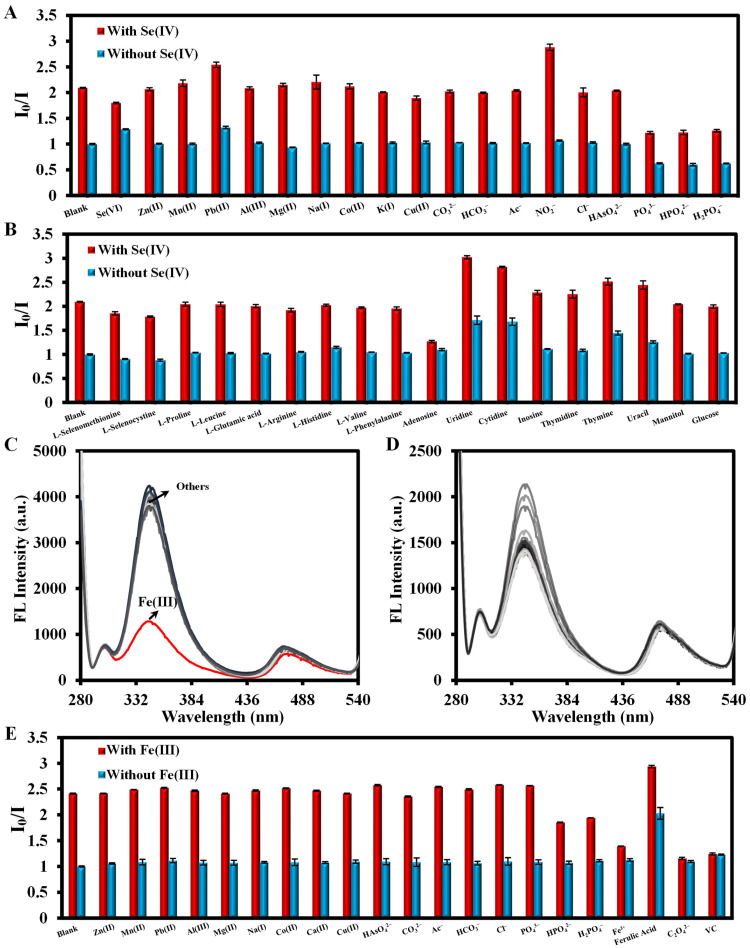
Selectivity and interference study of the ratiometric fluorescence method based on PCN-224-DABA for (**A**,**B**) Se(IV) and (**C**–**E**) Fe(III) detection.

To estimate the applicability of the developed method in the analysis of real samples, possible anions/cations, amino acids, and nucleosides were selected to investigate its selectivity and interference resistant properties. The results show that the synthesized PCN-224-DABA displays a better resistance to nucleoside analogs in the detection of Se(IV) (Figure 9) compared to the single DABA small molecule (Figure S8), but there was still a high interference for uridine and cytidine. It is important to highlight that the presence of alternative forms of Se, such as Se(VI), L-selenocystine, and L-selenomethionine, did not interfere significantly with the detection of Se(IV). Furthermore, there is a greater interference of the phosphate-like ions in detection, which is probably due to the presence of the TCPP porphyrin ligand in PCN-224-DABA (Figure 9A) [40]. For Fe(III) detection, Fe(II) as well as some reducing substances, such as oxalate and vitamin C (VC), can interfere with the course of redox reactions during the detection. Furthermore, when both Fe(III) and Se(IV) are presented in the detection system, they can be differentiated by the reaction time variation and the quenching ability between them. As shown in Figure S9, when the reaction time was 7 min, the same concentrations of Se(IV) and Fe(III) were added to the detection system. Only Fe(III) was observed to cause a change in the I_0_/I value, which can be eliminated by adding EDTA-2Na. When the reaction time was prolonged to 2 h, although Se(IV) could quench the fluorescence of PCN-224-DABA, its quenching degree was weaker than Fe(III). On the other hand, the addition of EDTA-2Na could no longer eliminate the effect of Fe(III) on the quenching of PCN-224-DABA fluorescence.

### 3.6. Real Sample Analysis

To validate the applicability of the PCN-224-DABA-based fluorescence method for the determination of Fe(III) and Se(IV) in real samples, spinach and selenium-enriched rice were selected as samples for the analysis, respectively. Figure S10A shows the fluorescence spectra of different systems, and the results demonstrate that the addition of EDTA-2Na causes some fluorescence recovery at 345 nm, which proves the existence of Fe(III) in spinach extract. The standard addition method was carried out on the extract and the Fe(III) content in spinach was calculated to be 4.730 mg/kg (Figure S10B), which is consistent with the results of AAS detection (5.565 mg/kg). The detection of Se content in selenium-enriched rice using PCN-224-DABA did not yield an accurate Se value in the sample, which was probably related to the complexity of the real samples, the loss of the element during the sample pretreatment process, and limitations of the sensitivity of the method. Subsequently, spiked recovery experiments were performed on selenium-enriched rice with recoveries of 83.4–116% and RSD values of less than 7.6%, indicating that the approach still has potential for the determination of Se content in real samples. In addition, the suitability of the assay for lake water samples was assessed. The recoveries and RSDs were determined by adding known concentrations of Se(IV) and Fe(III) solutions to the lake water samples. As shown in Table 3, the recoveries of Fe(III) are 82.0–111.8% and the spiked recoveries of Se(IV) are 96.3–115.6% with RSD values of less than 7.4%. In summary, the method presented in this manuscript demonstrates the potential to accurately quantify Se(IV) and Fe(III) in real samples.

## 4. Conclusions

In this paper, an innovative ratiometric fluorescent probe with multiple emissions was successfully synthesized through introducing DABA small molecules into a PCN-224 framework for the selective and sensitive detection of Se(IV) and Fe(III). The ratiometric fluorescence approach was constructed by fluorescence quenching at 345 nm in the presence of Se(IV) or Fe(III), but the intensity of fluorescence was unchanged at 475 nm. The Stern–Volmer equation of the analysis at different temperatures and the excitation spectra of PCN-224-DABA at different concentrations of Se(IV) and Fe(III) suggest a dynamic quenching mechanism. Zeta potentials indicate that the fluorescence quenching can be associated with a transfer of electrons between PCN-224-DABA and Se(IV)/Fe(III). FT-IR and XPS results show that five-membered cyclization and redox reactions took place between Se(IV) and PCN-224-DABA, and redox reactions between Fe(III) and PCN-224-DABA. In addition, the ratiometric fluorescent probe demonstrates good selectivity and interference resistance in the presence of other confounders, with LODs of 0.804 μM and 0.045 μM for Se(IV) and Fe(III), respectively. Finally, the established method provides satisfactory availability for the quantitative determination of Se(IV) and Fe(III) in spinach, selenium-enriched rice, and lake water, respectively. The ratiometric fluorescent probe developed in this study exhibits a limited linear response range for Se(IV) and Fe(III), and it lacks the capability to selectively identify both Se(IV) and Fe(III) simultaneously. This presents a constraint for its practical detection applications. Consequently, future research endeavors may be focused on enhancing the detection sensitivity and specificity of target substances through various strategies, including the modification of aptamer probes, the encapsulation of ion or molecule-imprinted materials, and the incorporation of enzyme-like materials.

## Figures and Tables

**Table 1:** Comparisons of different materials used for the detection of Se(IV).

Materials	Detection Ion	Liner Range (μM)	LOD (μM)	Ref.
AM + DAN	Se(IV)	0.063–15.829	2.03 × 10^−2^	[30]
OPD-CQDs	Se(IV)	1.000–100.000	8.60 × 10^−2^	[54]
DAB-CdTe@SiO_2_ QDs	Se(IV)	0–25.000	6.68 × 10^−3^	[56]
DAB-SiNPs	Se(IV)	12.660–126.600	2.41 × 10^−3^	[57]
CDs	Se(IV)	0.013–1.270	6.33	[58]
TGA-CdTe	Se(IV)	2.190–5.700	0.77	[59]
ABDO	Se(IV)	0.010–0.100	2.80 × 10^−3^	[60]
GAT	Se(IV)	10.000–50.000 50.000–100.000	1.70	[61]
HBTN-Se	Se(IV)	0–25.000	0.13	[62]
WHO	Se in water	–	0.13	[63]
DABA	Se(IV)	10.000–70.000 1.000–200.000	4.12 2.00	This work
PCN-224-DABA	Se(IV)	0.010–15	0.804	

AM: 9-Anthracenemethanol; DAN: 9-Anthracenemethanol; OPD: o-phenylenediamine; CQDs: carbon quantum dots; DAB: 3,3′-diaminobenzidine; QDs: quantum dots; CDs: carbon dots; TGA: thioglycolic acid; ABDO: 2-(2-(2-aminoethylamino)ethyl)-3′,6′-bis(ethylamino)-2′,7′-dimethylspiro[isoindoline-1,9′-xanthen]-3-one; GAT: gatifloxacin; HBTN: 1-Hydroxy-2-(benzothiazol-2-yl)naphthalene.

**Table 2:** Comparisons of different materials used for the detection of Fe(III).

Materials	Detection Ion	Liner Range (μM)	LOD (μM)	Ref.
Mg-Al-LDH-SA	Fe(III)	0.07–100.00	0.026	[49]
BCN	Fe(III)	0–3.00	0.185	[50]
MoSe_2_@Fe	Fe(III)	25.00–300.00	0.930	[52]
Cd-Cys NRs	Fe(III)	0.10–500.00	0.269	[64]
AgNCs	Fe(III)	0.02–50.00	0.010	[65]
^a^ CDs	Fe(III)	0–100.00	0.170	[66]
^b^ CDs	Fe(III)	1.00–100.00	0.300	[67]
^c^ CDs	Fe(II) Fe(III)	0–32.00 0–50.00	0.020 0.035	[68]
WHO	Fe(III)	–	5.36	[55]
EPA	Fe(III)	–	3.57	[12]
PCN-224-DABA	Fe(III)	0.01–4	0.045	This work

Mg-Al-LDH-SA: nano-structured Mg-Al layered double hydroxide intercalated with salicylic acid; BCN: graphitic carbon nitride (g-C3N4) doped with boron; Cys: cysteine; NRs: nanorods; AgNCs: silver nanoclusters. ^a^ CDs: carbon dots were synthesized using citric acid as the carbon precursors; ^b^ CDs: carbon dots were synthesized using chloroplasts as the carbon precursors; ^c^ CDs: carbon dots synthesized from citric acid and 1,10-phenanthroline.

**Table 3:** Determination of Fe(III) and Se(IV) in real samples.

Sample	Analyte	Detected (μM)	Found ^b^ (μM)	Spiked (μM)	Found ^b^ (μM)	Recovery ^c^ (%)	RSD (*n* = 3) (%)
Spinach	Fe(III)	5.565 ± 0.151 ^a^ mg/kg	4.730 ± 0.040 mg/kg				
Yun Lake		– ^d^	–	0.500	0.410	82.0	1.9
				1.000	0.837	83.7	4.4
				3.000	3.277	109.2	1.9
Jin Lake		–	–	0.500	0.423	84.7	2.5
				1.000	0.854	85.4	0.6
				3.000	3.352	111.8	1.6
Selenium-enriched rice	Se(IV)	–	–	1.000	1.160	116.0	3.0
				5.000	4.172	83.4	2.8
				10.000	9.365	93.6	7.6
Yun Lake		–	–	1.000	1.028	102.8	7.4
				5.000	5.340	106.8	6.9
				10.000	9.627	96.3	5.2
Jin Lake		–	–	1.000	1.135	113.5	3.6
				5.000	5.779	115.6	5.2
				10.000	10.577	105.8	5.4

^a^ Determined by AAS; ^b^ determined by PCN-224-DABA; ^c^ recovery (%) = (detected concentration − original concentration)/added concentration × 100%; ^d^ not presented in the sample or below the limit of detection of the method.

## Data Availability

Data are contained within the article and supplementary materials.

## Supplementary Materials

The following supporting information can be downloaded at: https://www.mdpi.com/article/10.3390/bios14120626/s1, Figure S1: (A and B) SEM, (C) TEM images, and (D) element mapping of PCN-224; Figure S2: SEM images of PCN-224-DABA with different TCPP/DABA ratios of (A) 1/1, (B) 1/3, (C)1/5, and (D) 1/9; Figure S3: Electron diffraction patterns of (A) PCN-224 and (B) PCN-224-DABA; (C) XRD spectra of PCN-224 and PCN-224-DABA; Figure S4: (A) The excitation and emission spectra of DABA and PCN-224-DABA; (B) fluorescence spectra of DABA, TCPP, and DABA + Se(IV), TCPP + Se(IV) at pH = 1.0, (C) fluorescence spectra of DABA, TCPP, DABA +Fe(III) and TCPP + Fe(III) at pH = 2.0; (D) UV/vis spectra of TCPP in different conditions; (E) Zeta potential of PCN-224-DABA at different pH; (F) zeta potential of (a) PCN-224-DABA with Tris-HCl (pH = 1.0), (b) PCN-224-DABA with Se(IV), (c) PCN-224-DABA with Tris-HCl (pH = 2.0), and (d) PCN-224-DABA with Fe(III); Figure S5: (A) Fluorescence spectra of PCN-224-DABA with different TCPP/DABA ratios, and (B) their corresponding histograms; (C) histogram of ratios at different PCN-224-DABA dilutions ; Figure S6: The emission spectra of (A) DABA, (B) PCN-224-DABA, and (C) PCN-224 at different pH; (D) storage stability of PCN-224-DABA at pH = 1.0 and pH = 2.0; Figure S7: Influence of DABA concentration (A) and pH (B) on the Se(IV) detection; effect of temperature (C) and reaction time (D) on the Se(IV) detection at pH = 1; effect of temperature (E) and reaction time (F) on the Se(IV) detection at pH = 5; (G) fluorescence spectra of DABA with different Se(IV) concentrations and (H) scatter and (I) linear plot of F0/F versus Se(IV) concentration at pH = 1.0; (J) fluorescence spectra of DABA with different Se(IV) concentrations and (K) scatter and (L) linear plot of F/F0 versus Se(IV) concentration at pH = 5.0; Figure S8: Selectivity of the fluorescence method based on DABA at (A) pH = 5.0 and (B) pH = 1.0. The concentrations of analytes are 100 μM; Figure S9: Histograms of scaled fluorescence values for different reaction systems; Figure S10: (A) Fluorescence spectra of different reaction systems and (B) standard addition curve for Fe(III) detection in spinach.
