# Supplementary material for: A Ratiometric Fluorescence Method Based on PCN-224-DABA for the Detection of Se(IV) and Fe(III)
Source: Biosensors (Basel). 2024 Dec 19;14(12):626. doi: 10.3390/bios14120626 (PMC11675067; doi:10.3390/bios14120626)
Supplement: Supplementary file 1 [file biosensors-14-00626-s001.zip › biosensors-3311314-supplementary.pdf]

Supplementary Material

A ratiometric fluorescence method based on PCN-224-DABA for the detection of Se(IV) and Fe(III)

Mao-Ling Luo [1], Guo-Ying Chen [1], Wen-Jia Li [2], Jia-Xin Li [1], Tong-Qing Chai [1], Zheng-Ming Qian [2,*], Feng-Qing Yang [1,*]

[1] School of Chemistry and Chemical Engineering, Chongqing University, Chongqing 401331, China; 20185486@cqu.edu.cn (M.-L.L); 20221801017@stu.cqu.edu.cn (G.-Y.C.); liwenjia@hec.cn (W.–J.L.); 202218021016@stu.cqu.edu.cn (J.-X.L); 20175531@cqu.edu.cn (T.-Q.C.)

[2] Dongguan HEC Cordyceps R&D Co., Ltd, Dongguan, Guangdong 523850, China

* Correspondence: fengqingyang@cqu.edu.cn (F.-Q.Y.); qianzhengming@hec.cn (Z.-M.Q.)

Supplementary Methods

1. Chemicals and materials

Tetrakis (4-carboxyphenyl) porphyrin (TCPP, 97%), L-Selenomethionine (98%), and cobalt (II) chloride hexahydrate ($CoCl_2·6H_2O$, 99%) were obtained from Shanghai Macklin Biochemical Technology Co., Ltd. (Shanghai, China). Manganese chloride tetrahydrate ($MnCl_2·4H_2O$, 99.5%), sodium acetate (CH_3COONa, 99%), and sodium nitrite ($NaNO_2$, 99.99%) were purchased from Shanghai Aladdin Biochemical Technology Co., Ltd (Shanghai, China). Potassium phosphate (K_3PO_4, 98%) was obtained from Shanghai Yuanye Biotechnology Co., Ltd. (Shanghai, China). Sodium selenite ($Na_2SeO_3·5H_2O$, ≥98%), ferric chloride ($FeCl_3$, 99%), and sodium arsenate ($Na_2HAsO_4·7H_2O$, ≥98%) were purchased from Beijing MREDA Technology Co., Ltd. (Being, China). Zirconyl chloride octahydrate ($ZrOCl_2·8H_2O$, ≥99.0%) was obtained from Shanghai Dibo Technology Co., Ltd. (Shanghai, China). The 3,4-Diaminobenzoic acid (DABA, 97%), selenic acid (H_2SeO_4, 40% aqueous solution), and copper(II) sulfate ($CuSO_4·5H_2O$, 99%) were purchased from Shanghai Meryer Biochemical Technology Co., Ltd. (Shanghai, China). L-selenocystine (98%) was purchased from Shanghai ACMEC Biochemical Technology Co., Ltd. (Shanghai, China). Trometamol (Tris, 99.99%) was obtained from BBI Life Science Corporation Co., Ltd. (Shanghai, China). Adenosine (99%), cytidine (99%), inosine (99%), adenosine (99%), uridine (99%), thymine (99%), and uracil(99%) were obtained from Sigma-Aldrich Lab &

Production Material Co., Ltd. (Shanghai, China). Aluminum nitrate ($Al(NO_3)_3$, ≥99.0%), glucose, benzoic acid (BA, ≥99.5%), lead nitrate ($Pb(NO_3)_2$, ≥99.0%), magnesium sulfate ($MgSO_4 \cdot 7H_2O$, ≥99.0%), sodium chloride (NaCl, ≥99.5%), potassium chloride (KCl, ≥99.5%), ascorbic Acid (AA, ≥99.7%), benzoic acid (BA, ≥99.5%), ethylenediaminetetraacetic acid disodium salt (EDTA-2Na, ≥99.0%), sodium hydrogen carbonate ($NaHCO_3$, ≥99.5%), N, N-dimethylformamide (DMF), hydrochloric acid (HCl), and nitric acid (HNO_3) were obtained from Chengdu Chron Chemical Co., Ltd. (Chengdu, China). L-proline (99%) was purchased from Biotopped Life Science Co., Ltd. (Being, China). L-histidine (99%) was purchased from Chengdu Huaxia Chemical Reagent Co., Ltd. (Chengdu, China). D-Mannitol (＞98%) was obtained from PUSH Biotechnology Co., Ltd. (Chengdu, China). Zinc acetate dihydrate ($(CH_3COO)_2Zn \cdot 2H_2O$, ≥99.0%), potassium dihydrogen phosphate (NaH_2PO_4, ≥99.5%), disodium hydrogen phosphate (Na_2HPO_4, ≥99%), sodium oxalate ($Na_2C_2O_4$, ≥99.8%), sodium hydroxide (NaOH, ≥98.0%), and sodium carbonate (Na_2CO_3, ≥99.8%) were purchased from Shanghai Titan Scientific Co., Ltd. (Shanghai, China). Calcium dichloride ($CaCl_2$, ≥96.0%) was obtained from DAMAO Chemical Reagent Factory (Tianjin, China). Ferrous sulfate ($FeSO_4$, 98%) was obtained from Shanghai Rhawn Chemical Technology Co., Ltd. (Shanghai, China). L-leucine, L-glutamic acid, L-arginine, and L-valine were purchased from Targetmol Chemicals Inc. L-phenylalanine (99.5%) was obtained from Shanghai Sangon Biotechnology Co., Ltd. (Shanghai, China).

Ferulic acid (98%) was purchased from Chengdu Phytochemical Pure Biotechnology Co., Ltd. (Chengdu, China). Thymidine was obtained from Biosharp Life Science Co., Ltd. (Beijing, China). All substances were dissolved in Tris-HCl at pH = 1.0 or pH = 2.0.

2. Instrumentation

The optical characteristics of PCN-224-DABA were probed by an F-7100 fluorescence spectrophotometer (Hitachi Ltd., Tokyo, Japan). Temperature control using DF-101S collector-type constant temperature heating magnetic stirrer (Gongyi Yuhua Instrument Equipment Co., Ltd., Zhengzhou, China) and smart thermostatic water bath (Changsha Mitr Instrument Equipment Co., Ltd., Changsha, China). Fourier transform infrared (FT-IR) spectroscopy was conducted on a Nicolet iS10 spectrometer (Shimadzu, Japan). The sample's X-ray diffraction (XRD) patterns were acquired through an X′ pert Powder diffractometer (Malvern Panalytical Ltd., the Netherlands) with secondary beam graphite monochromated Cu Ka radiation. Transmission electron microscopy (TEM) images and element distribution analysis were recorded using a Talos F200S (Thermo Fisher Scientific Inc. Czech Republic) electron microscope working at 200 kV equipped with energy dispersive X-ray spectrometer (EDX). For analysis of the surface composition and chemical states of the product, X-ray photoelectron spectroscopy (XPS) spectra were obtained using a Thermo Fisher K-Alpha instrument (Thermo Fisher Scientific, USA). The scanning electron microscopy (SEM) images of PCN-224-DABA

were obtained through a JSM-7600F field-emission SEM (JEOL Ltd., Tokyo, Japan). Zeta potential at different pH was determined using NanoBrook Omni (Brookhaven Instrument, UK). All the pH of the solution was measured by an FE28 pH meter (Mettler-Toledo Instruments, Shanghai, China). The ultrasonic cleaner used in this study was obtained from Jiangsu Kunshan Jielimei Ultrasonic Instrument Co., Ltd. (Kunshan, China). The ultrapure water prepared through a water purification system (ATSelem 1820A, Antesheng Environmental Protection Equipment, Chongqing, China) was used for all the experiments.

3. Pre-treatment of real samples

For the extraction of Fe(III) from spinach, the procedure was referred to GB5009.90-2016 "Determination of iron in food", the section of dry digestion. In short, 1.000 g of spinach sample (purchased from Yonghui Supermarket) was accurately weighed and heated in a crucible over low heat, carbonized until no smoke was produced, and then transferred to a muffle furnace and ashed at 550 °C for 4 h and cooled to room temperature. Subsequently, 1 mL of HNO_3 (v:v, 1:1) was added to the dry ashed spinach sample, transferred to a 10 mL volumetric flask, and the inner jar and the inner lid were washed with a small amount of ultrapure water for 2–3 times, and the washed solution was combined and then mixed. After the pH of the above specimens was adjusted to 2 with solid NaOH, they were treated by solid phase extraction and filtered through 0.22 μm membrane filter. Moreover, a reagent blank was prepared as

described above.

The extraction method of selenium was referred to the wet digestion method listed in GB5009.93-2017 with slight modifications. In brief, accurately weighed 3.0 g of rice sample, 10 mL of nitric acid-perchloric acid (v/v: 9/1) and a few glass beads were added, and then was covered with the surface dish for cold digestion overnight. On the next day, the solution was heated on a hot plate, and continue to heat it until the remaining volume was about 2 mL when the solution became clear and colorless and accompanied by white smoke, during which a small amount of HNO_3 was added if the solution turns black. The solution was cooled to room temperature and then 5 mL of HCl (6 M) was added and continued to be heated until it became clear and colorless with white smoke to a remaining volume of about 2 mL, at which Se (VI) was reduced to Se (IV). Subsequently, after cooling to room temperature, 6 mL of water was added and the pH was adjusted to weakly acidic (pH 4–6) with solid NaOH. Finally, the volume was fixed with Tris-HCl (pH = 1.0) to 100 mL. Moreover, a reagent blank was prepared as described above.

4. Se(IV) detection with DABA

As shown in Figure S7A, 1 mM of DABA was selected for Se(IV) detection. Firstly, the fluorescence emission peak of DABA shifts from 345 nm to 380 nm at pH ≥ 4.0. Additionally, Se(IV) quenches the fluorescence at pH < 4 and enhances it at pH ≥ 4. Therefore, pH levels of 1.0 and 5.0 were selected for the following experiments (Figure S7B). The effects of reaction temperature and

time on the assay were examined sequentially at two pH conditions as the magnitude of F_0/F at pH=1.0 and F/F_0 at pH=5.0, in which F_0 and F are the fluorescence intensity of DABA in the absence and presence of Se(IV), respectively. The final assay conditions were as follows: for pH = 1.0, 100 μL DABA (pH = 1.0, 1 mM), 800 μL Tris-HCl (pH = 1.0), and 100 μL Se (IV) (pH = 1.0, 1 mM) were added sequentially, and the reaction was placed in a 70 °C water bath for 10 min; for pH = 5.0, 100 μL DABA (pH = 5.0, 1 mM), 800 μL tris-HCl (pH = 5.0), and 100 μL Se (IV) (pH = 5.0, 1 mM) were kept in 30 °C for 10 min. Finally, the detection of Se(IV) was linear in the range of 1–200 μM with a detection limit of 2.00 μM at pH = 1.0 and in the range of 10–70 μM with a detection limit of 4.12 μM at pH = 5.0.

Supplementary figures

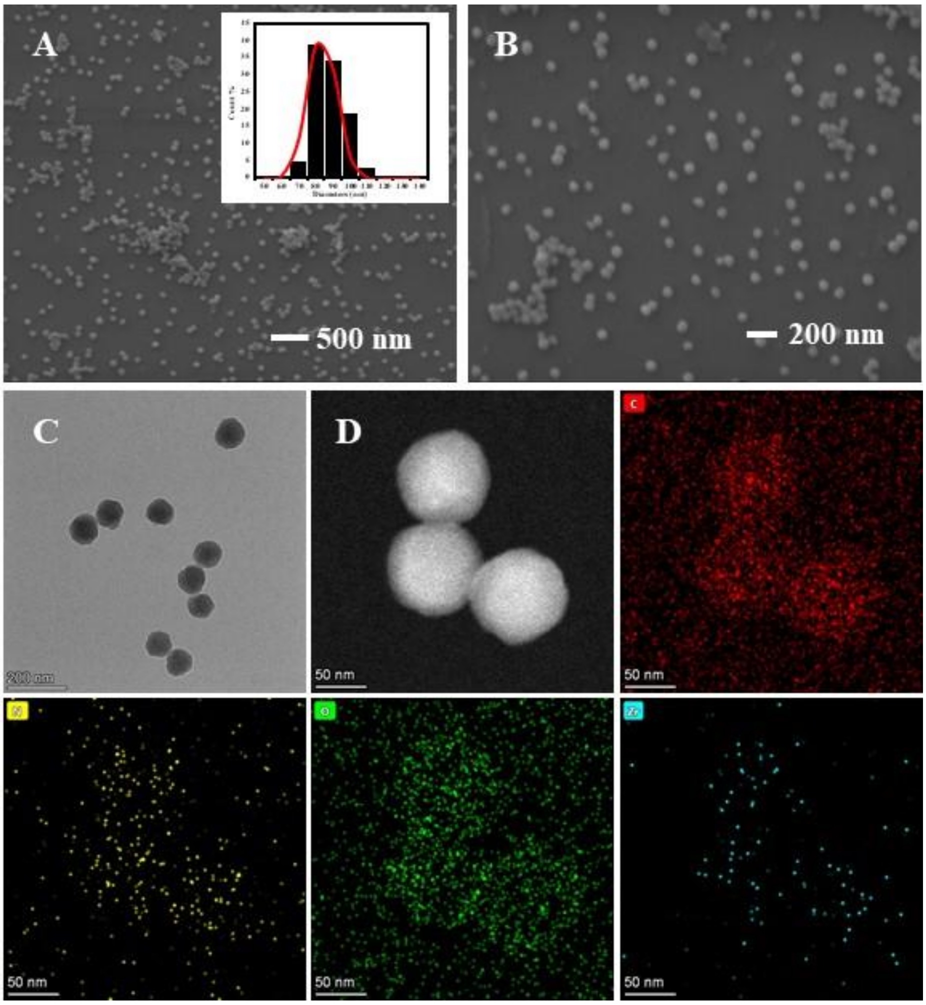

Figure S1. (A & B) SEM, (C) TEM images, and (D) element mapping of PCN-224.

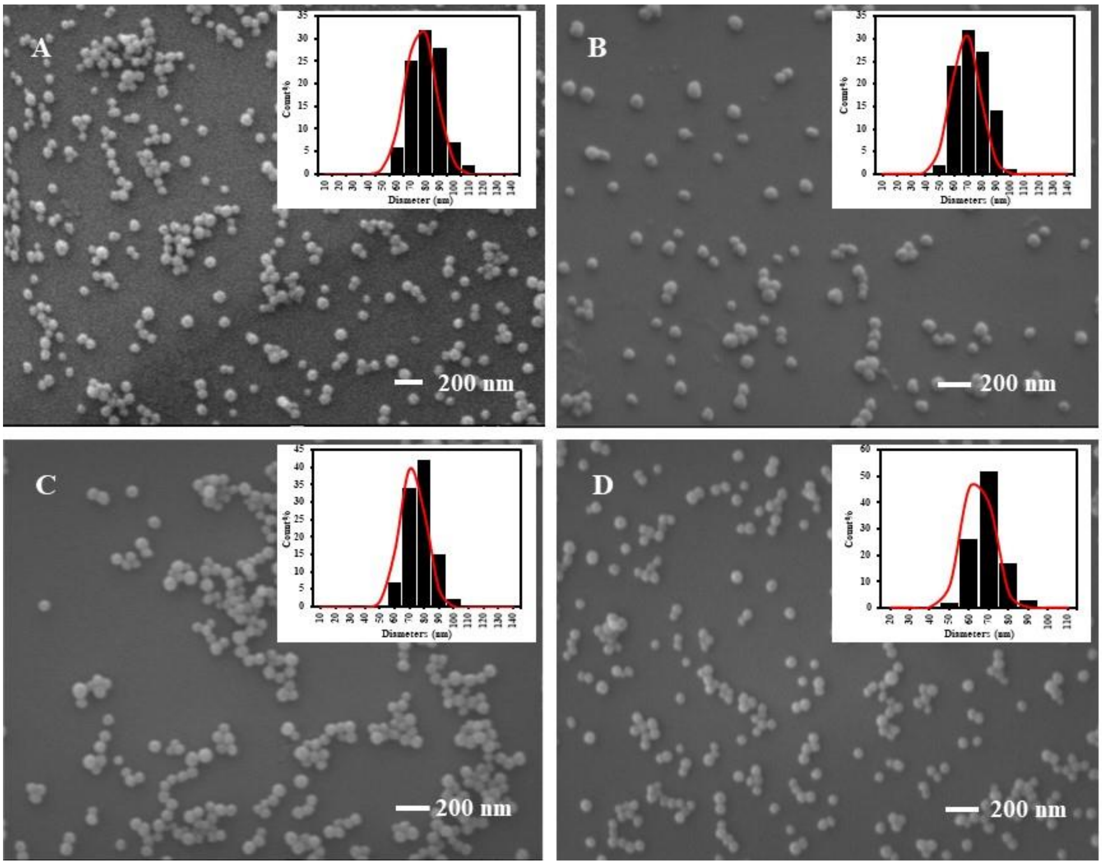

Figure S2. SEM images of PCN-224-DABA with different TCPP/DABA ratios of (A) 1/1, (B) 1/3, (C)1/5, and (D) 1/9.

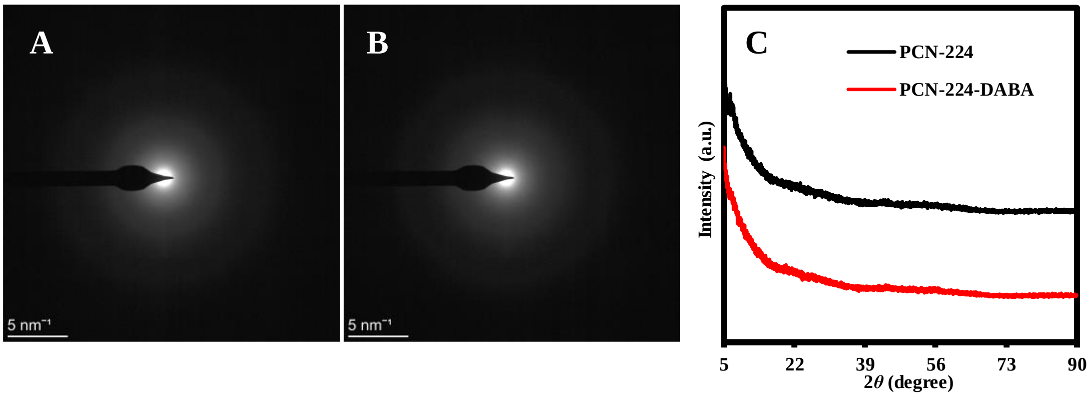

Figure S3. Electron diffraction patterns of (A) PCN-224 and (B) PCN-224-DABA; (C) XRD spectra of PCN-224 and PCN-224-DABA.

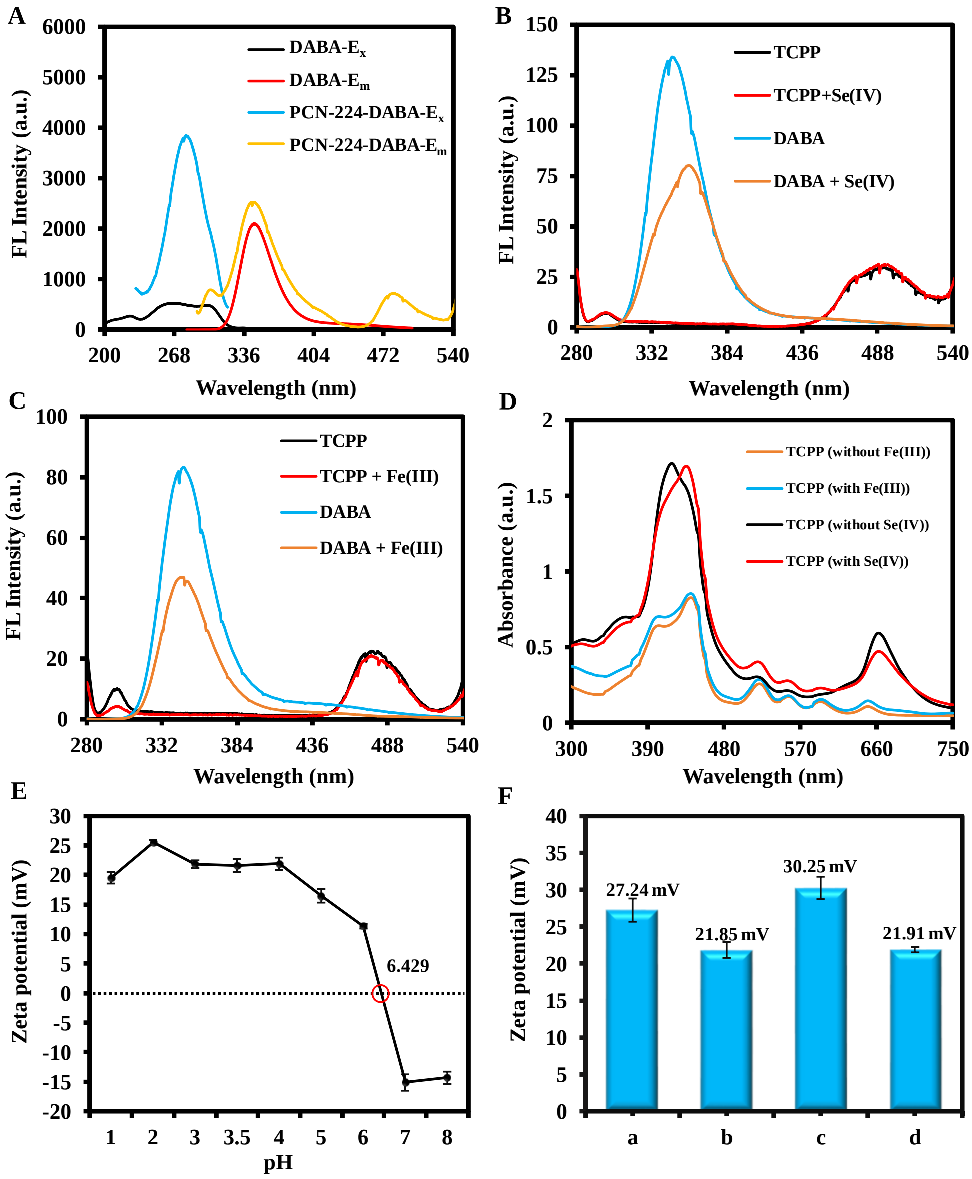

Figure S4. (A) The excitation and emission spectra of DABA and PCN-224-DABA; (B) fluorescence spectra of DABA, TCPP, and DABA + Se(IV), TCPP + Se(IV) at pH = 1.0, (C) fluorescence spectra of DABA, TCPP, DABA +Fe(III) and TCPP + Fe(III) at pH = 2.0; (D) UV/vis spectra of TCPP in different conditions; (E) Zeta potential of PCN-224-DABA at different pH; (F) zeta potential of (a) PCN-224-DABA with Tris-HCl (pH = 1.0), (b) PCN-224-DABA with Se(IV), (c) PCN-224-DABA with Tris-HCl (pH = 2.0), and (d) PCN-224-DABA with Fe(III).

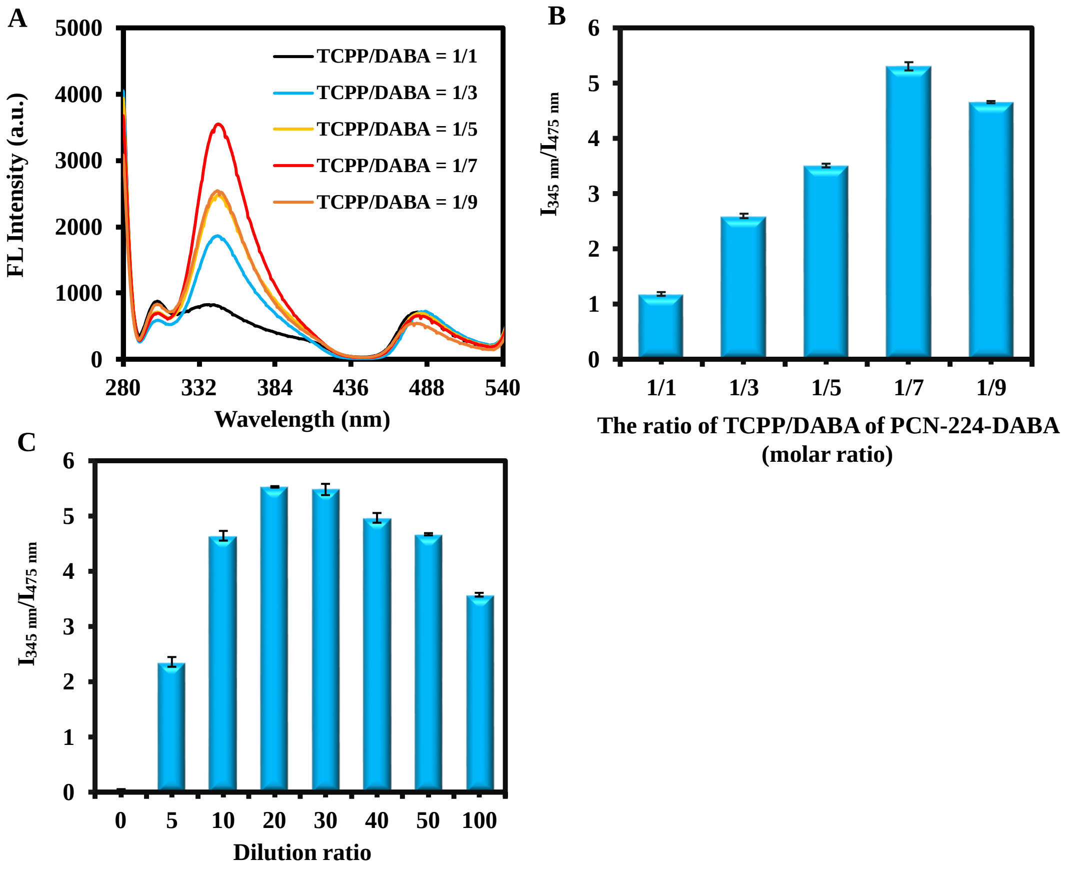

Figure S5. (A) Fluorescence spectra of PCN-224-DABA with different TCPP/DABA ratios, and (B) their corresponding histograms; (C) histogram of ratio at different PCN-224-DABA dilutions.

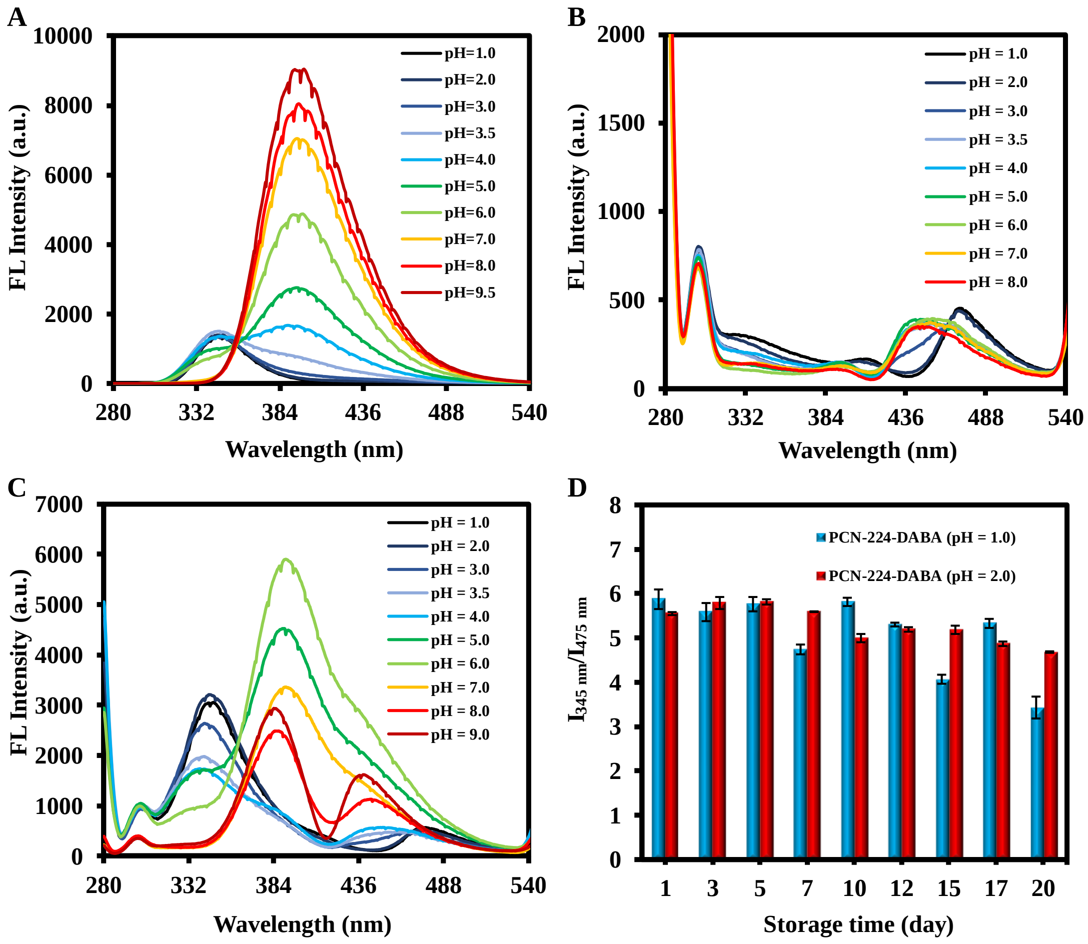

Figure S6. The emission spectra of (A) DABA, (B) PCN-224-DABA, and (C) PCN-224 at different pH; (D) storage stability of PCN-224-DABA at pH=1.0 and pH=2.0.

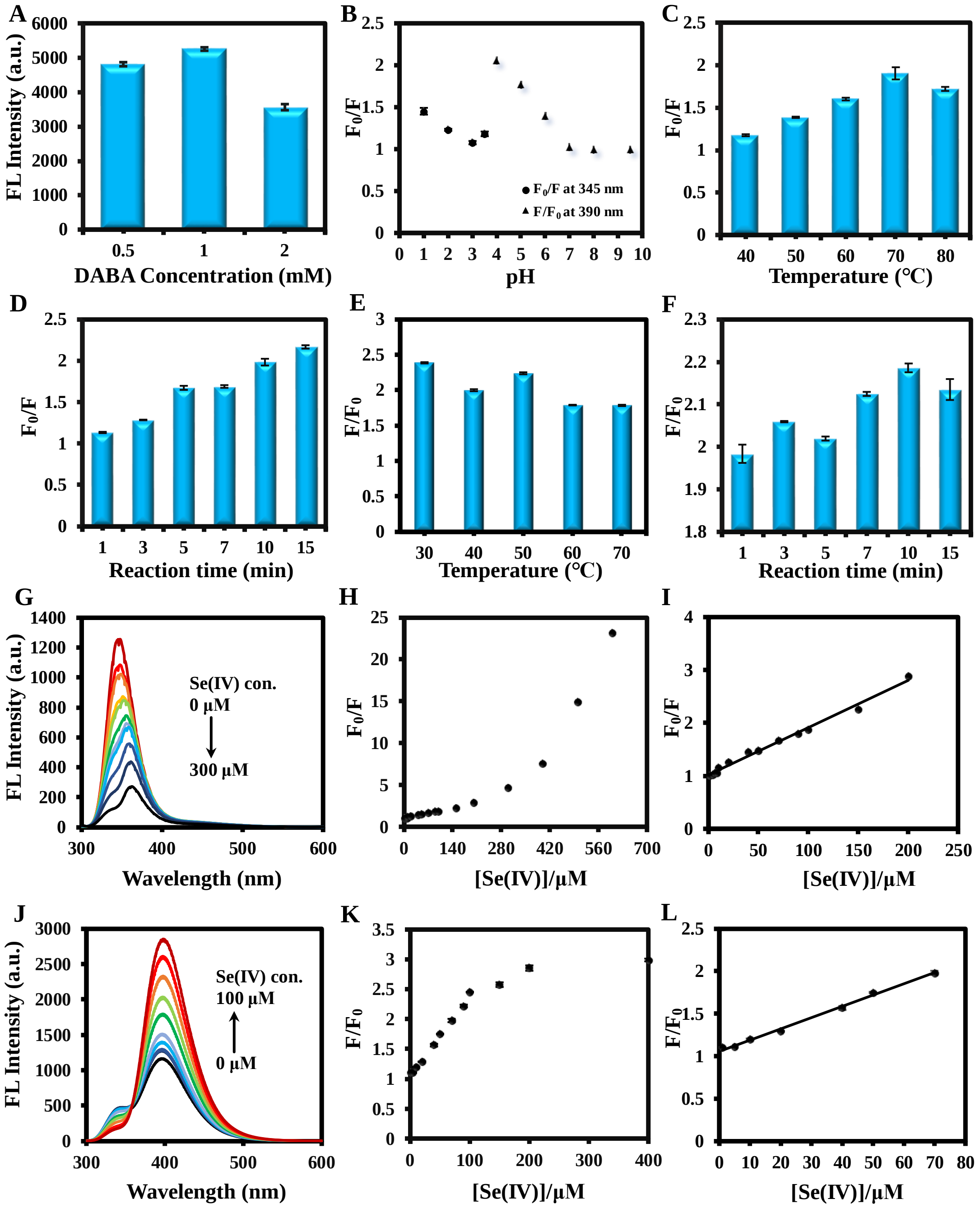

Figure S7. Influence of DABA concentration (A) and pH (B) on the Se(IV) detection; effect of temperature (C) and reaction time (D) on the Se(IV) detection at pH = 1; effect of temperature (E) and reaction time (F) on the Se(IV) detection at pH = 5; (G) fluorescence spectra of DABA with different Se (IV) concentrations and (H) scatter and (I) linear plot of F_0/F versus Se (IV) concentration at pH = 1.0; (J) fluorescence spectra of DABA with different Se (IV) concentrations and (K) scatter and (L) linear plot of F/F_0 versus Se (IV) concentration at pH = 5.0.

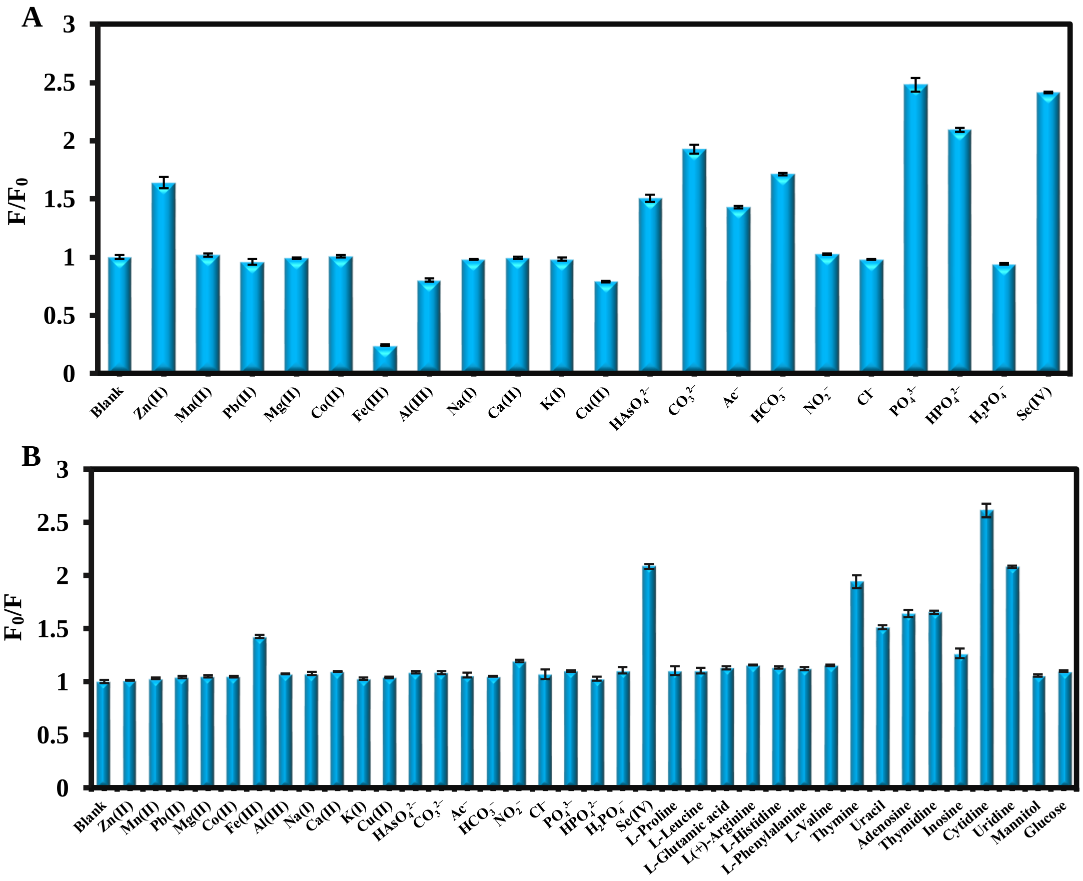

Figure S8. Selectivity of the fluorescence method based on DABA at (A) pH = 5.0 and (B) pH = 1.0. The concentrations of analytes are 100 μM.

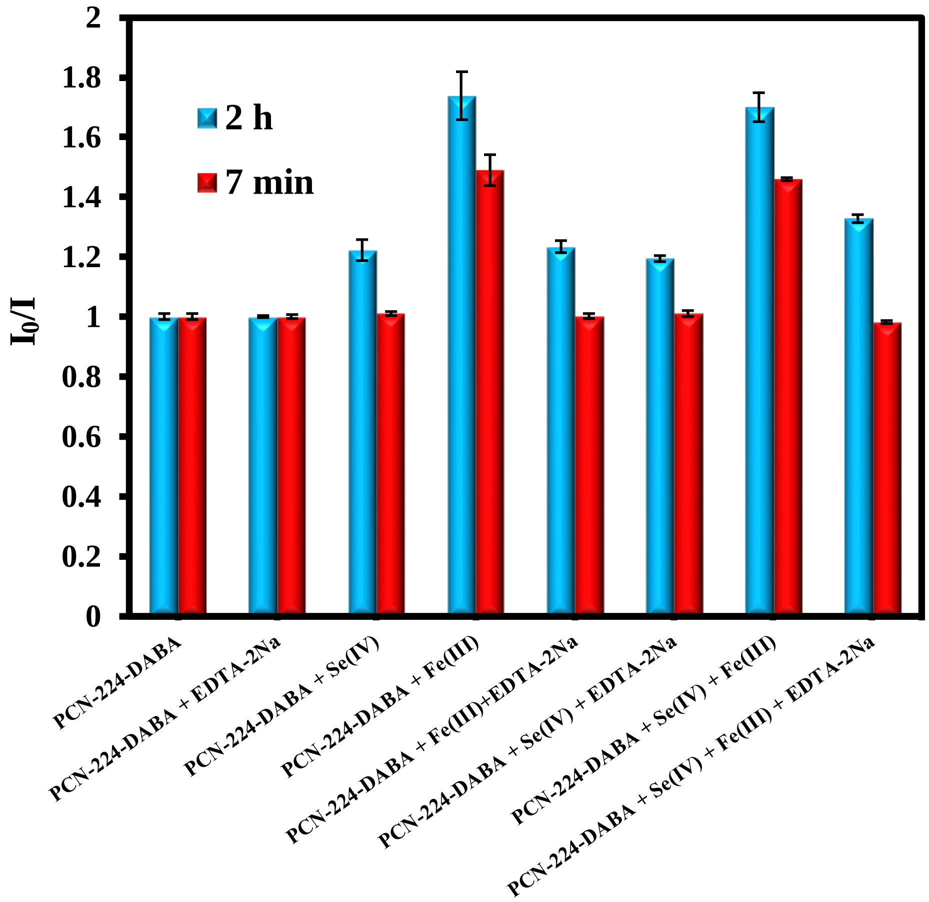

Figure S9. Histograms of scaled fluorescence values for different reaction systems.

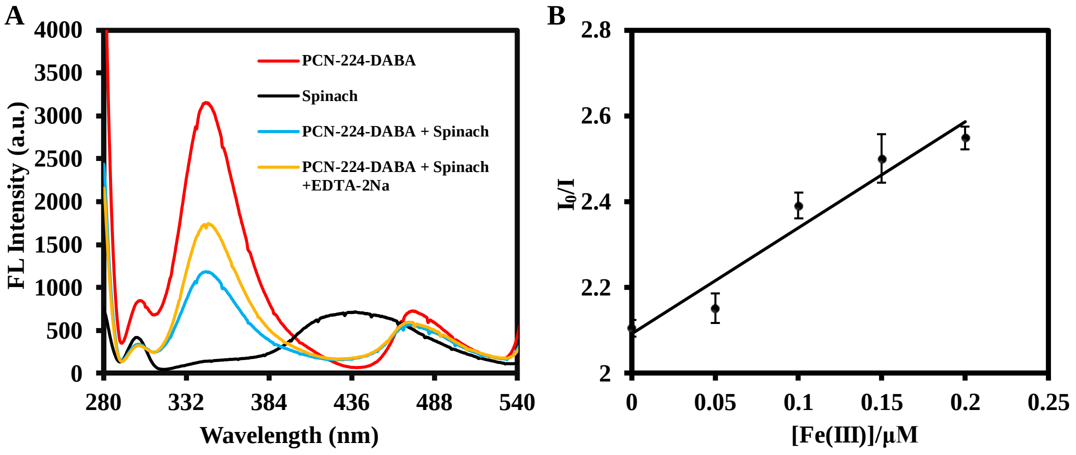

Figure S10. (A) Fluorescence spectra of different reaction systems and (B) standard addition curve for Fe(III) detection in spinach.
